# *In Vitro* Immunomodulation of a Whole Blood IFN-γ Release Assay Enhances T Cell Responses in Subjects with Latent Tuberculosis Infection

**DOI:** 10.1371/journal.pone.0048027

**Published:** 2012-10-29

**Authors:** Rajiv L. Gaur, Megan M. Suhosk, Niaz Banaei

**Affiliations:** 1 Department of Pathology, Division of Infectious Diseases and Geographic Medicine, Stanford University School of Medicine, Stanford, California, United States of America; 2 Department of Medicine,Division of Infectious Diseases and Geographic Medicine, Stanford University School of Medicine, Stanford, California, United States of America; 3 Clinical Microbiology Laboratory, Stanford Hospital and Clinics, Palo Alto, California, United States of America; Hopital Raymond Poincare - Universite Versailles St. Quentin, France

## Abstract

**Background:**

Activation of innate immunity via pathogen recognition receptors (PRR) modulates adaptive immune responses. PRR ligands are being exploited as vaccine adjuvants and as therapeutics, but their utility in diagnostics has not been explored. Interferon-gamma (IFN-γ) release assays (IGRAs) are functional T cell assays used to diagnose latent tuberculosis infection (LTBI); however, novel approaches are needed to improve their sensitivity.

**Methods:**

In vitro immunomodulation of a whole blood IGRA (QuantiFERON®-TB GOLD In-Tube) with Toll-like receptor agonists poly(I:C), LPS, and imiquimod was performed on blood from subjects with LTBI and negative controls.

**Results:**

In vitro immunomodulation significantly enhanced the response of T cells stimulated with *M. tuberculosis* antigens from subjects with LTBI but not from uninfected controls. Immunomodulation of IGRA revealed T cell responses in subjects with LTBI whose T cells otherwise do not respond to in vitro stimulation with antigens alone. Similar to their *in vivo* functions, addition of poly(I:C) and LPS to whole blood induced secretion of inflammatory cytokines and IFN-α and enhanced the surface expression of antigen presenting and costimulatory molecules on antigen presenting cells.

**Conclusions:**

In vitro immunomodulation of whole blood IGRA may be an effective strategy for enhancing the sensitivity of T cells for diagnosis of LTBI.

## Introduction

There are ∼2 billion individuals worldwide with latent tuberculosis infection (LTBI) [1]. Because each latent infection carries a 5–10% lifetime risk of progressing to active disease, LTBI comprises a significant reservoir for future TB epidemics [2], [3]. Treatment of LTBI is a proven strategy for preventing progression of LTBI to active disease [4]. Epidemic modeling studies suggest that eradication of LTBI in high burden countries is necessary for successful elimination of TB [5], [6]. Accurate diagnosis of LTBI is therefore a pivotal component of TB control programs.

Diagnosis of LTBI is made through measuring cell-mediated immune responses to *M. tuberculosis* antigens and excluding active disease. The tuberculin skin test (TST) has served this purpose for more than a century but it lacks specificity due to cross reaction with the *M. bovis* BCG vaccine and environmental mycobacteria [7], [8]. More recently, the interferon-gamma (IFN-γ) release assays (IGRAs) were developed for *in vitro* diagnosis of LTBI [9], [10]. Performed by stimulating patient blood cells *in vitro*, currently available commercial IGRAs measure IFN-γ released from antigen-specific T cells upon stimulation with *M. tuberculosis* antigens that are absent from BCG and most nontuberculous mycobacteria thus contributing to increased specificity. The QuantiFERON®-TB GOLD In-Tube assay (QFT-GIT, Cellestis Limited, Carnegie, Victoria, Australia) is a commercial IGRA that measures IFN-γ response as the difference in IFN-γ concentration when blood is incubated with TB antigen stimulation in a TB Antigen tube minus the concentration when incubated without antigen in the Nil tube. The test is interpreted based on predefined cut-off values [10]. IGRAs are attractive alternatives to TST for their improved specificity, pre- and post-analytical standardization, and logistical advantages [10]. However, similar to TST, they lack sensitivity for detection of latent (range, 40% to 100%) or active (range, 83% to 90%) infection [9], [11]. The sensitivity of IGRAs is further compromised in high-risk groups such as HIV infected individuals, immunocompromised hosts, and children [12], [13]. In addition, IGRAs lack reproducibility in cohorts undergoing serialized testing, with within-subject variability ranging from 16 to 80% [14].

The poor performance of IGRAs is in part due to the low assay cut-off values, close to the detection limit of IFN-γ. Attempts to improve the sensitivity of IGRAs have focused on addition of novel T cell antigens or simultaneous measurement of IFN-γ and biomarkers downstream of IFN-γ signaling pathway [15], [16]. These modifications have yielded marginal improvements, and novel approaches are needed to further enhance the sensitivity and reproducibility of IGRAs.

Pathogen-associated molecular patterns (PAMPs) are conserved microbial products with immunomodulatory properties [17], [18]. During infection, PAMPs are recognized by the innate immune cells via several families of pathogen recognition receptors (PRRs) of which the Toll-like receptor (TLR) family is best characterized [17], [18]. Activation of pathogen sensors triggers intracellular signaling pathways which culminate in the expression and release of inflammatory cytokines such as interleukin 6 (IL-6) and 12 (IL-12) and type I interferons (IFN-α/β) [17], [18]. These mediators in turn stimulate the maturation of antigen presenting cells and initiation of adaptive immune responses such as the development and proliferation of antigen-specific effector T cell subsets [19]–[21]. In the case of intracellular pathogens, effector T cells egress from lymph nodes and migrate to the site of infection where they activate infected macrophages via IFN-γ [22]. Some studies suggest PAMPs also enhance the function of effector T cells [23]. *M. tuberculosis* stimulates PRRs through a number of TLR ligands and other PAMPs [24]–[28]. Studies in humans and mice have implicated TLR2, TLR9, and TLR signaling molecules in susceptibility to TB [17], [29].

Because of their immunomodulatory properties, PRR ligands are being exploited as adjuvants in vaccine formulations and as therapeutics for infectious, autoimmune, and neoplastic disorders [30]–[33]. In this report we investigated whether *in vitro* immunomodulation of QFT-GIT with TLR agonists polyinosine-polycytidylic acid (poly(I:C); TLR3), lipopolysaccharide (LPS; TLR4), and imiquimod (IMQ; TLR7) can be used to enhance the response of T cells in individuals with LTBI. We also investigated the potential mechanisms through which TLR agonists modulate IGRA.

## Materials and Methods

### Ethics Statement

A written informed consent was obtained from volunteers. This study was approved by the Stanford University Institutional Review Board.

### Study subjects

Healthy adults were recruited from the Stanford Hospital clinical laboratories. After obtaining consent, volunteers were asked to complete a standardized questionnaire to assess established risk factors for TB exposure [10]. LTBI was defined as prior history of positive TST (≥10 mm in immunocompetent and ≥5 mm in immunocompromised hosts) and QFT-GIT and ≥1 risk factor but with no signs or symptoms of active TB. Uninfected control was defined as US born with prior negative TST and QFT-GIT and no risk factors. The TST was performed more than three years prior to current experiments while the QFT-GIT has been performed annually since 2007. IGRA-unresponsive individuals were defined as subjects with a history of LTBI based on positive TST and QFT-GIT but with a negative QFT-GIT result during this study. Volunteers meeting inclusion criteria were enrolled and studied consecutively. Some volunteers were recruited the second time for the mechanistic studies (Table S1).

### Reagents

PRR ligands Poly(I:C), LPS and IMQ were purchased from InvivoGen (San Diego, CA). IL-6 and IL-12 were purchased from R&D System (Minneapolis, MN) and IFN-α was purchased from PBL Interferon Source (Piscataway, NJ). All compounds were reconstituted in endotoxin-free water and stored at −70°C. Anti-human CD45 antibody was purchased from Invitrogen (Carlsbad, CA). All other antibodies were purchased from BD Biosciences (San Jose, CA).

### QuantiFERON-TB Gold In-Tube assay

The QFT-GIT assay was performed with fresh blood according to package insert with some modification to accommodate addition of immunomodulators. Up to 10 µl of each agonist or an equivalent volume of endotoxin-free water was added to each Nil and TB Ag tube. Blood was collected in a 10 ml Kendall Monoject Green Stopper tube and 1 ml was quickly transferred to each QFT-GIT tube. Tubes were then mixed and placed in a 37°C incubator for 22 hours or as indicated. ELISA was performed according to package insert. The remaining plasma was stored at −70°C. Calculation of IFN-γ concentrations was done with the software provided by the manufacturer. TB Antigen tubes with IFN-γ>10 IU/ml were diluted in PBS to determine the exact value. The effect of immunomodulators on IFN-γ response was calculated using the following formula: modulated response = [(TB Ag plus immunomodulator) minus (Nil tube plus immunomodulator)].

### Dose response curve

Indicated concentrations of each agonist or endotoxin-free water was added to each Nil tube. The tubes were then mixed and placed in a 37°C incubator for 22 hours. The IFN-γ concentrations were measured as described above.

### Cytokine profiling assay

The cytokine profiling assay was performed at the Human Immune Monitoring Center (HIMC) at Stanford University. 100 µl of plasma from the QFT-GIT Nil tube was subjected to cytokine profiling. Procarta Cytokine Assay custom 51-plex kit (Affymetrix, Palo Alto, CA) was used according to manufacturer's recommendations.

### Flowcytometry

The QFT-GIT tubes were incubated upside down to prevent migration of cells into the gel portion of the tube. At the end of 3 hours incubation blood was transferred to a 15 ml polypropylene tube containing 100 µl of 20 mM EDTA. Samples were vortexed vigorously twice for 30 seconds with an interim incubation at ambient temperature for 15 minutes. 9 ml of 1× FACS™ Lysing Solution (BD Biosciences) was added per tube. Samples were incubated at room temperature for 10 minutes and directly transferred to a −70°C freezer for storage. The cell surface immune profiling studies were performed at the HIMC. The cells were thawed in warm media, washed once with FACS buffer (PBS supplemented with 2% FBS and 0.1% sodium azide) and resuspended at 1×10^7^ viable cells/ml. 5×10^5^ cells per well were stained for 45 minutes at ambient temperature with the following anti-human antibodies: CD11c (561352), CD14 (560180), CD19 (561295; 560177), CD40 (555591), CD80 (560925), CD86 (555657), CD123 (558714), HLA-DR (560651), HLA ABC (561346) and CD45 [MHCD4530]. BD Biosciences catalog numbers are indicated in parenthesis and Invitrogen catalog number is indicated in brackets. Cells were washed three times with FACS buffer, and resuspended in 200 µl FACS buffer. At least 200,000 cells per sample were collected using DIVA 6.0 software on an LSRII flow cytometer (BD Biosciences). Data analysis was performed using FlowJo v9.3 by gating on live cells based on forward versus side scatter profiles, then on singlets using forward scatter area versus height, followed by cell subset-specific gating.

### Statistical analysis

Statistical analyses were performed using the Prism software (GraphPad, San Diego, CA). A non-parametric test, the Wilcoxon signed-rank test of medians, was used to compare responses with and without TLR ligands. All statistical tests were computed for a two-sided type I error rate of 5%.

## Results

### TLR agonists enhance IFN-γ response in whole blood IGRA

**Figure 1:**
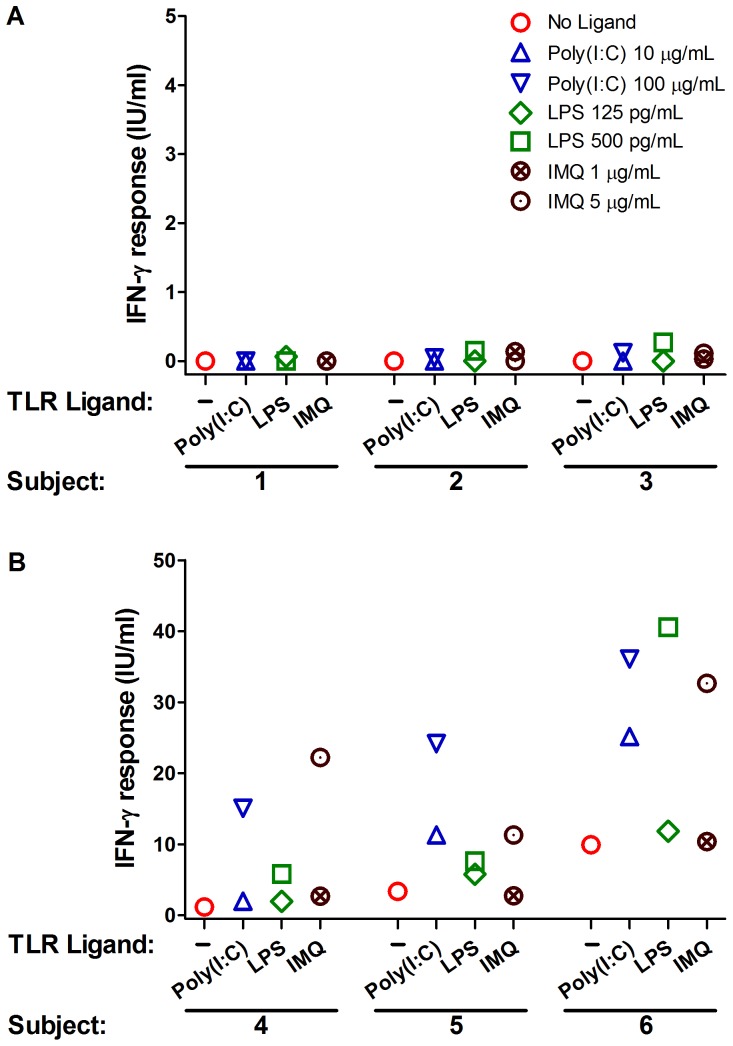
Immunomodulation of Quantiferon assay enhances the IFN-γ response in subjects with LTBI. Representative IFN-γ response (TB Ag minus Nil) for uninfected controls (Panel A) and individuals with LTBI (Panel B) tested with the QFT-GIT assay in the absence or presence of two concentrations of poly(I:C), LPS, and imiquimod (IMQ). Representative individuals from each group showed similar results as the rest of the group in terms of the trend and the intensity of response. Data are representative of eight individuals in each group.

**Figure 2:**
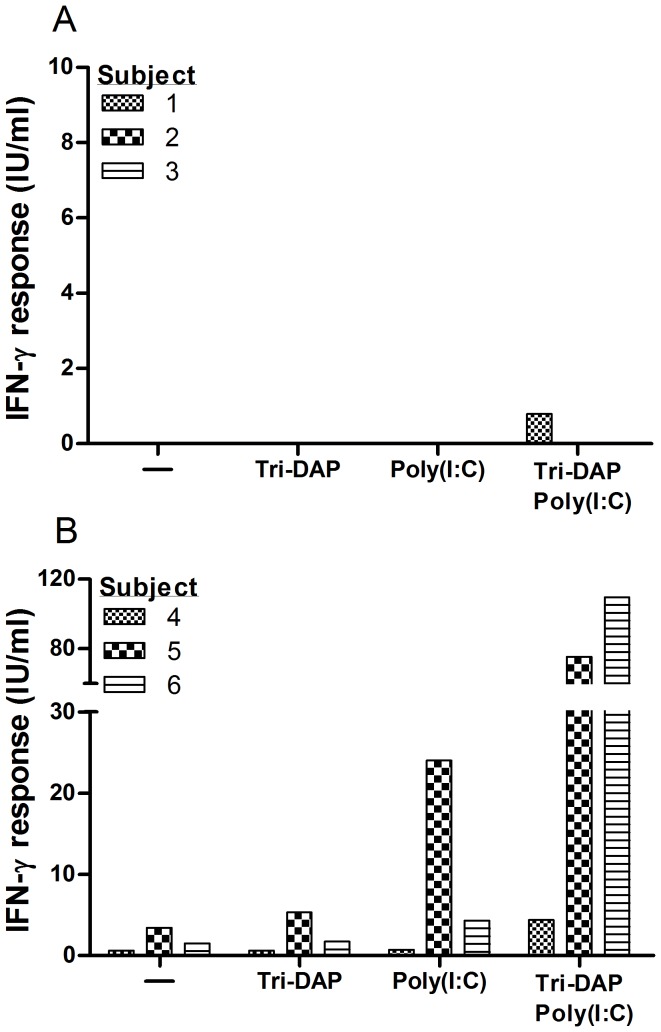
Poly(I:C) and Tri-DAP synergize for immunomodulation of Quantiferon assay in subjects with LTBI. Representative IFN-γ response (TB Ag minus Nil) for uninfected controls (Panel A) and individuals with LTBI (Panel B) tested with the QFT-GIT assay in the absence or presence of poly(I:C) 10 µg/ml and Tri-DAP 10 µg/ml. Representative individuals from each group showed similar results as the rest of the group in terms of the trend and the intensity of response. Data are representative of six individuals in each group.

To test whether immunomodulation of whole blood IGRA with PRR ligands can enhance the response of *M. tuberculosis*-specific T cells, QFT-GIT assay was performed in 8 individuals with LTBI and 8 uninfected controls in the absence and presence of TLR agonists poly(I:C), lipopolysaccharide (LPS), and imiquimod. These TLR agonists were chosen because their corresponding receptors (TLR3, TLR4, and TLR7, respectively) are differentially expressed by antigen presenting cell subsets in the peripheral blood and because intracellular signaling through these T helper 1-polarizing TLRs spans the signaling pathways downstream of all TLRs [17], [34]. The concentration of each agonist was chosen from a dose response curve performed in the Nil tube (Figure S1). Compared to the standard assay, modulation of QFT-GIT with poly(I:C) (10 and 100 µg/ml), LPS (125 and 500 pg/ml), and imiquimod (1 and 5 µg/ml) resulted in a dose-dependent enhancement of IFN-γ response in blood from subjects with LTBI but not from the uninfected controls (Figure 1 and Table 1). Higher concentrations of LPS (10 ng/ml) elicited a non-specific response in T cells from uninfected controls (data not shown). Testing of 10 additional subjects with LTBI and 10 additional uninfected controls with an intermediate concentration of poly(I:C) (40 µg/ml), LPS (250 pg/ml), and imiquimod (2 µg/ml) showed a significant enhancement of IFN-γ response in T cells from subjects with LTBI but not from the uninfected controls (Figure S2). Although LPS at a concentration of 250 pg/ml significantly increased the IFN-γ response from uninfected controls, the magnitude of the responses remained low and did not exceed 0.5 IU/ml (Figure S2). Overall, the effect of immunomodulation on IGRA with each ligand was heterogeneous across infected subjects with T cells from each individual responding variably to different concentrations of the same stimulus. The enhancement of IFN-γ response ranged from 1.6 to 12.1 fold (median, 5.7) with poly(I:C), 1 to 9.5 fold (median, 3.1) with LPS, and 1.4 to 9.1 fold (median, 3) with imiquimod (Figure S2). There was also variability in the magnitude of IFN-γ response enhancement when immunomodulation was repeated in the same individual on two different occasions (data not shown). We also investigated for synergy between PAMPs based on studies in mice showing synergistic action between TLRs and other PAMP sensors such as Nod-like receptor family member, Nod1, in generation of adaptive immune responses [35]. Immunomodulation of QFT-GIT with a combination of poly(I:C) (10 µg/ml) and Nod1 agonist Tri-DAP (10 µg/ml) was highly synergistic compared to each ligand alone in T cells from six individuals with LTBI while a low response was elicited in T cells from 2 of 6 individuals in the control group (Figure 2).

**Table 1:** Comparison of modulated and unmodulated QFT-GIT results from infected subjects and uninfected controls.

Treatment	No LTBI		LTBI		
	Median	P value*	Median	P value*	P value‡
**No PAMP**	0		4.6		<0.005
**Poly(I:C) - 10**	0	1.0	11.9	0.02	<0.005
**Poly(I:C) - 100**	0	0.5	60.5	0.02	<0.005
**LPS - 0.125**	0	0.5	8.8	0.03	<0.005
**LPS - 0.5**	0	0.63	24.6	0.03	<0.005
**IMQ - 1**	0	1.0	7.6	0.05	<0.005
**IMQ - 5**	0.14	0.13	32.7	0.02	<0.005

* Comparison of PAMP to No PAMP.

‡ Comparison of LTBI to No LTBI.

### Modulation of IGRA elicits IFN-γ responses in IGRA-unresponsive subjects

**Figure 3:**
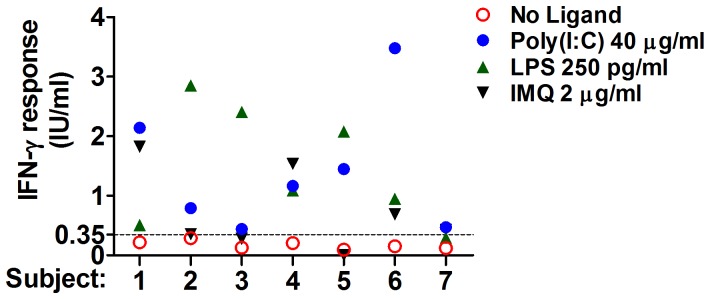
Immunomodulation of Quantiferon assay elicits an IFN-γ response in IGRA-unresponsive subjects with LTBI. IFN-γ response (TB Ag minus Nil) for individuals with history of LTBI. Each individual was tested with the QFT-GIT assay in the absence or presence of poly(I:C) 40 µg/ml, LPS 250 pg/ml, and imiquimod (IMQ) 2 µg/ml. The cut-off value for the standard QFT-GIT assay (dashed line) is shown for reference.

The findings above suggest that immunomodulation of IGRA may be a useful strategy for revealing T cell responses in subjects with LTBI who otherwise do not respond to *in vitro* stimulation with antigens alone. To test this hypothesis, we performed the QFT-GIT assay without and with immunomodulation in seven individuals with documented histories of LTBI (positive TST and QFT-GIT) but currently with negative QFT-GIT results due to spontaneous positive to negative reversions. Modulation of QFT-GIT with poly(I:C) (40 µg/ml), LPS (250 pg/ml), and imiquimod (2 µg/ml) increased the IFN-γ responses in all of these individuals compared to the standard assay (P<0.05) (Figure 3). The extent of modulation with each TLR agonist was variable in blood from each subject; certain individuals tended to respond better to certain modulators. Overall, LPS was most consistent in eliciting a response greater than the cutoff value for the standard assay.

### TLR agonists enhance markers of innate immune activation in whole blood IGRA

**Figure 4:**
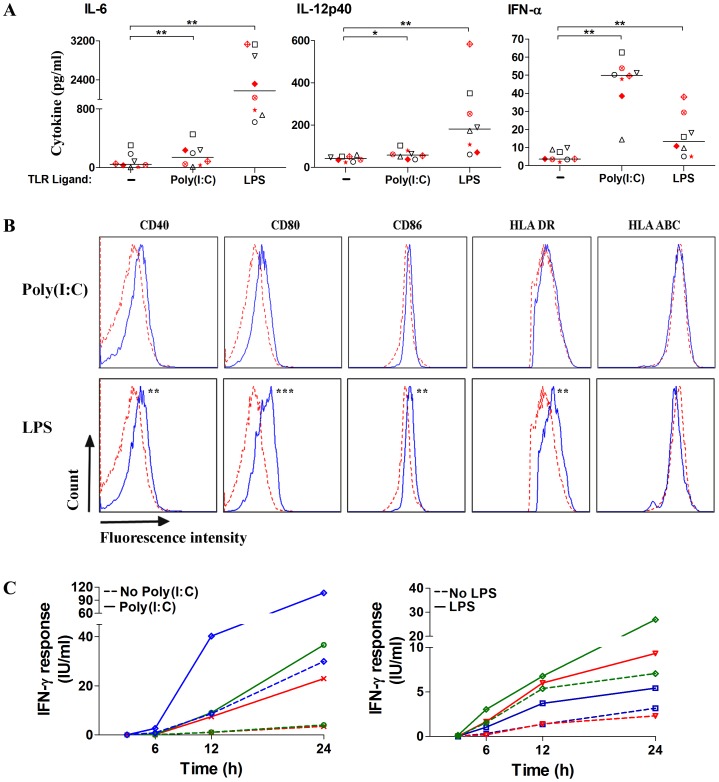
Immunomodulation of Quantiferon assay with TLR ligands enhances markers of innate immune activation. (Panel A) Induction of IL-6, IL-12, and IFN-α in whole blood stimulated with TLR agonists. Blood from four donors with LTBI (red symbols) and four uninfected controls (black symbols) was incubated in the QFT-GIT Nil tube in the absence or presence of poly(I:C) 40 µg/ml and LPS 250 pg/ml for 22 h. (Panel B) Flow cytometry analysis of surface expression of MHC class I and II and costimulatory molecules on monocytes stimulated with poly(I:C) and LPS. Whole blood from six donors was incubated in the QFT-GIT Nil tube in the absence (dashed red line) or presence (solid blue line) of poly(I:C) 40 µg/ml and LPS 250 pg/ml for 3 h. (Panel C) Kinetics of IFN-γ response (IFN-γ response, TB Ag minus Nil) in the QFT-GIT assay without and with immunomodulation with poly(I:C) 40 µg/ml and LPS 250 pg/ml. Data in B and C are representative of 6 individuals in each group. The Wilcoxon signed-rank test was used to compare responses with and without PRR ligands. The asterisks indicate significant difference. *, *P*≤0.05, **, *P*≤0.005, *** *P*≤0.0005.

We hypothesized that enhancement of IFN-γ response in the whole blood IGRA by TLR agonists is mediated through the same inflammatory cytokines and type I interferons that mediate initiation of adaptive immunity *in vivo* through activation of innate immunity [17]. Therefore, we used a multiplex immunoassay to measure 51 cytokines in the Nil tube of whole blood QFT-GIT from four healthy and four infected donors in the presence or absence of poly(I:C) and LPS immunomodulation. The levels of IL-6, IL-12 p40, IFN-α, and other cytokines were significantly increased after stimulation of blood cells with poly(I:C) (40 µg/ml) and LPS (250 pg/ml) (Figure 4A and Table S2). However, immunomodulation of QFT-GIT assay with purified IL-6 at 200 and 2000 pg/ml, IL-12 at 12.5 and 25 pg/ml, and IFN-α at 15 and 30 pg/ml, alone or in combination, was not sufficient to recapitulate the effects of TLR agonists on the QFT-GIT assay (data not shown). To determine whether immunomodulation with TLR agonists also enhanced the maturation of antigen presenting cells in the QFT-GIT assay, we use flowcytometry to measure the surface expression of antigen presenting molecules (MHC class I and II) and costimulatory molecules (CD40, CD80, and CD86) in monocytes (CD14+), B cells (CD19+), and dendritic cells of myeloid (CD11c+) or plasmocytoid (CD123+) lineage in six donors. Compared to unstimulated blood cells, treatment with LPS (250 pg/ml) for 3 hours, significantly increased the surface expression of MHC II and costimulatory molecules in monocytes and some of them in B cells and dendritic cells (Figure 4B and Figure S3). Immunomodulation of whole blood with poly(I:C) (40 µg/ml) had a partial enhancement of surface expression of MHC II and costimulatory molecules in antigen presenting cells (Figure 4B and Figure S3). The induction of cytokines and maturation of antigen presenting cells with poly(I:C) and LPS correlated with earlier and more robust release of IFN-γ in the QFT-GIT assay (Figure 4C).

## Discussion

In this report we describe a novel application for PRR agonists as *in vitro* immunomodulators of IGRA for diagnosis of latent *M. tuberculosis* infection. The findings from this study suggest that immunomodulation of IGRA with PRR ligands may be a useful strategy for addressing the shortcomings in the sensitivity of the commercially available whole blood IGRA [9], [11], [14]. Although immunomodulation was shown to be effective in a small cohort of infected subjects of Asian, Indian, and Caucasian ancestries, longitudinal studies with a larger number of participants and in more diverse populations are needed to determine the sensitivity and specificity of IGRA with immunomodulators at various cutoffs compared to the standard assay. Immunomodulation of IGRA may be particularly useful in the pediatric and immunocompromised populations where IGRAs have had lower sensitivities [12], [13]. It was recently shown that IFN-γ response of T cells stimulated with *M. tuberculosis* whole cell lysate (WCL) compared with purified secreted antigens better correlated with lower risk of subsequent HIV-associated TB [36]. Given the abundance of potent PAMPs in the *M. tuberculosis* WCL, it would be interesting to determine whether immunomodulation of IGRA with PAMPs elicits an IFN-γ response that better correlates with immunity to TB. *In vitro* immunomodulation may also have broader applications for sensitive diagnosis of infectious diseases and autoimmune disorders with T cell-mediated pathogenesis [37]–[39]. Whether immunomodulation of IGRA is equally effective for eliciting a T cell response in subjects with active TB was not investigated in this study. It will be interesting to determine whether immunomodulators can overcome the functional impairment of “exhausted” T cells that occur during chronic infection [40].

The modulation of T cell responses in IGRA by exogenous PAMPs raises the question whether endogenous signals modulate the output of IGRA and therefore account for the within-subject variability that has been often observed with IGRA [14]. The heterogeneous T cell responses observed between and within subjects in this study suggest endogenous biological factors present in blood cooperate with exogenous TLR ligands to modulate IGRA. It was recently shown that the antimycobacterial activity of human monocyte-derived macrophages depends on whether autologous or heterologous plasma is used during differentiation and activation of the macrophages [41]. These findings are consistent with our observations and altogether suggest that the presence of certain endogenous immunomodulators in blood can impact the *in vitro* function of antigen presenting cells in IGRA. Within-subject variability poses a major dilemma for the clinician who must decide how to manage patients with reversion or conversion of their IGRA results [14]. Studies on temporal association between human conditions (infection and inflammation) and/or behavior (biological and social) and IGRA test results are needed to investigate the source of endogenous immunomodulatory signals. Elimination of variation in endogenous signals could enhance the reproducibility and accuracy of IGRA.

The mechanism by which TLR agonists elicited an earlier and more robust IFN-γ response from antigen-specific T cells in the whole blood IGRA is not known. The induction of inflammatory cytokines and IFN-α by poly(I:C) and LPS are consistent with the known *in vivo* properties of TLR agonists as initiators of adaptive immune responses through stimulation of innate immunity [17], [18]. However, immunomodulation of QFT-GIT assay with purified IL-6, IL-12, and IFN-α, alone or in combination, was insufficient to recapitulate the effects of TLR agonists suggesting that other factors may be essential. The differential regulation of antigen presenting molecules (MHC class II) and costimulatory molecules in antigen presenting cells stimulated with LPS and poly(I:C) suggests that these TLR agonists may be enhancing T cell responses in the QFT-GIT assay through different mechanisms. The finding that Nod1 agonist Tri-DAP synergizes with TLR3 agonist poly(I:C) in enhancing the IGRA response to *M. tuberculosis* antigens is consistent with animal studies showing a synergistic action between Nod1 and TLR agonists for priming of Th1 immune responses [35]. The cellular origin of cytokines and the relative contribution of T cell subsets to IFN-γ release remains to be determined [34]. Analogous to vaccine strategies where PRR ligands are formulated to elicit desired protective immune responses, modulation of IGRA with PRR ligands may be an *in vitro* strategy to elicit responses from T cell subsets with prognostic value [31], [42], [43].

In summary, we showed that *in vitro* immunomodulation of IGRA with TLR agonists is a novel strategy for eliciting T cell responses in individuals infected with *M. tuberculosis*. A deeper understanding of the cellular and molecular basis of immunomodulation of whole blood IGRA is necessary to reap their full potential.

## Supporting Information

Figure S1 **Whole blood IFN-γ dose response to TLR agonists.** The concentration of IFN-γ in whole blood treated with indicated concentrations of poly(I:C) (Panel A), LPS (Panel B), and imiquimod (IMQ) (Panel C) in Nil tubes is shown. Data from nine individuals is shown.
(TIF)

Figure S2 **Immunomodulation of Quantiferon assay enhances the IFN-γ response in subjects with LTBI.** IFN-γ response (TB Ag minus Nil) for ten uninfected controls (Panel A–C) and ten individuals with LTBI (Panel D–F) tested with the QFT-GIT assay in the absence or presence of poly(I:C) 40 µg/ml (Panel A,D), LPS 250 pg/ml (Panel B,E), and imiquimod (IMQ) 2 µg/ml (Panel C,F). Panel G shows comparison of modulated and unmodulated QFT-GIT results from infected and uninfected subjects. The Wilcoxon signed-rank test of medians, was used to compare paired modulated and unmodulated results from infected and uninfected subjects.
(TIF)

Figure S3 **Flow cytometry analysis of surface expression of MHC and costimulatory molecules on B cells and dendritic cells stimulated with poly(I:C) and LPS.** Whole blood was incubated in the QFT-GIT Nil tube in the absence (dashed red line) or presence (solid blue line) of poly(I:C) 40 µg/ml (Panel A) and LPS 250 pg/ml (Panel B) for 3 h. mDC, myeloid dendritic cell; pDC, plasmacytoid dendritic cell. The Wilcoxon signed-rank test was used to compare responses with and without PRR ligands. The asterisks indicate significant difference. *, *P*≤0.05, **, *P*≤0.005, *** *P*≤0.0005.
(TIF)

Table S1 **Characteristics of study subjects and the experiments they participated in.**
(XLSX)

Table S2 **Cytokine profile of whole blood in QFT-GIT Nil tube stimulated with TLR agonists.** Blood was incubated in the QFT-GIT Nil tube in the absence or presence of poly(I:C) (40 µg/ml) and LPS (250 pg/ml) for 22 h. Cytokine profiling was performed with Procarta Cytokine Assay custom 51-plex kit. Median values in pg/ml for eight individuals (four latently infected individuals and four healthy controls) are shown.
(XLSX)
