# Supplementary figures and images for: In Vitro Immunomodulation of a Whole Blood IFN-γ Release Assay Enhances T Cell Responses in Subjects with Latent Tuberculosis Infection
Source: PLoS One. 2012 Oct 29;7(10):e48027. doi: 10.1371/journal.pone.0048027 (PMC3483295; doi:10.1371/journal.pone.0048027)

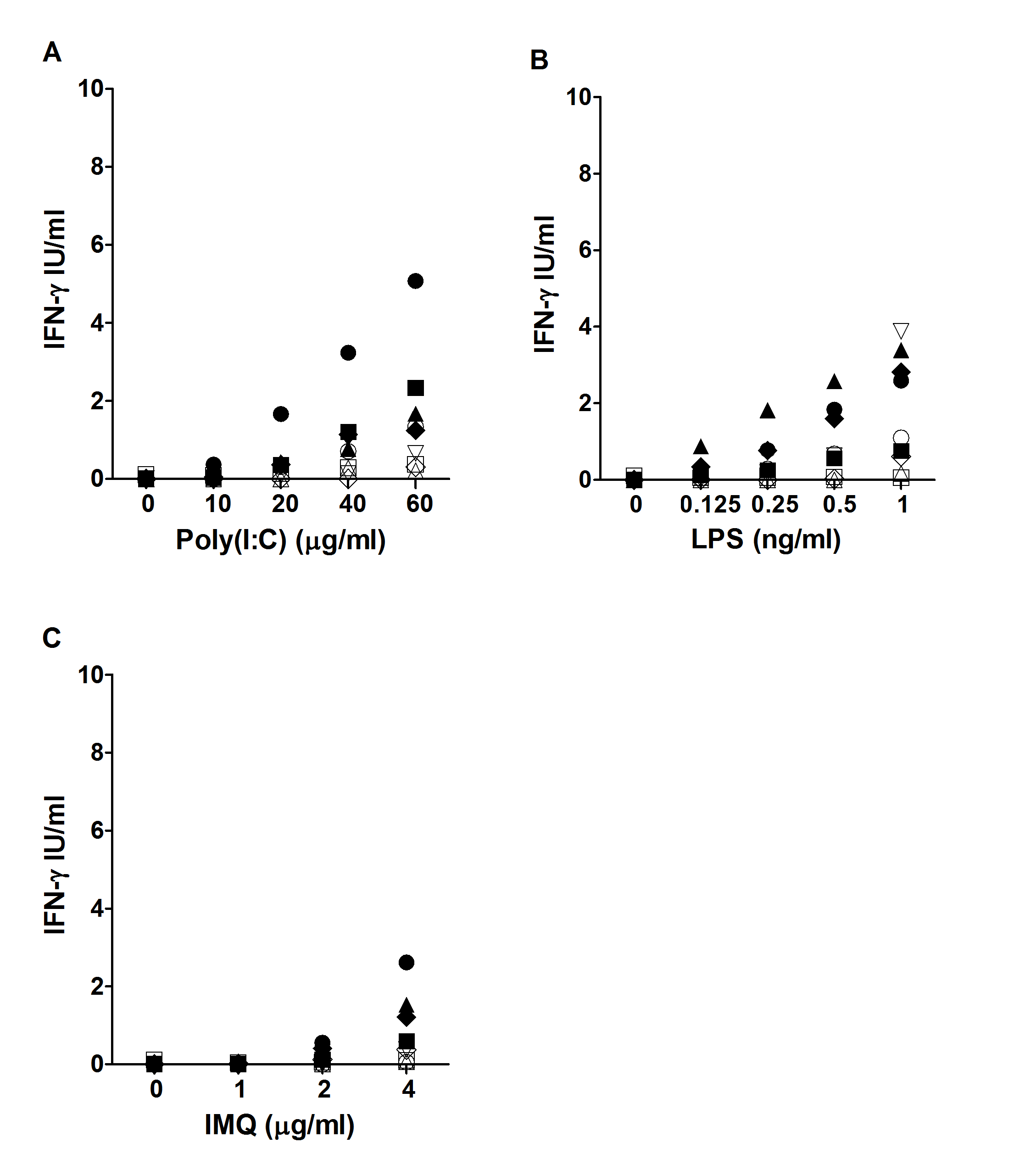

Supplement: Figure S1 — Whole blood IFN-γ dose response to TLR agonists. The concentration of IFN-γ in whole blood treated with indicated concentrations of poly(I:C) (Panel A), LPS (Panel B), and imiquimod (IMQ) (Panel C) in Nil tubes is shown. Data from nine individuals is shown. (TIF) [file pone.0048027.s001.tif]

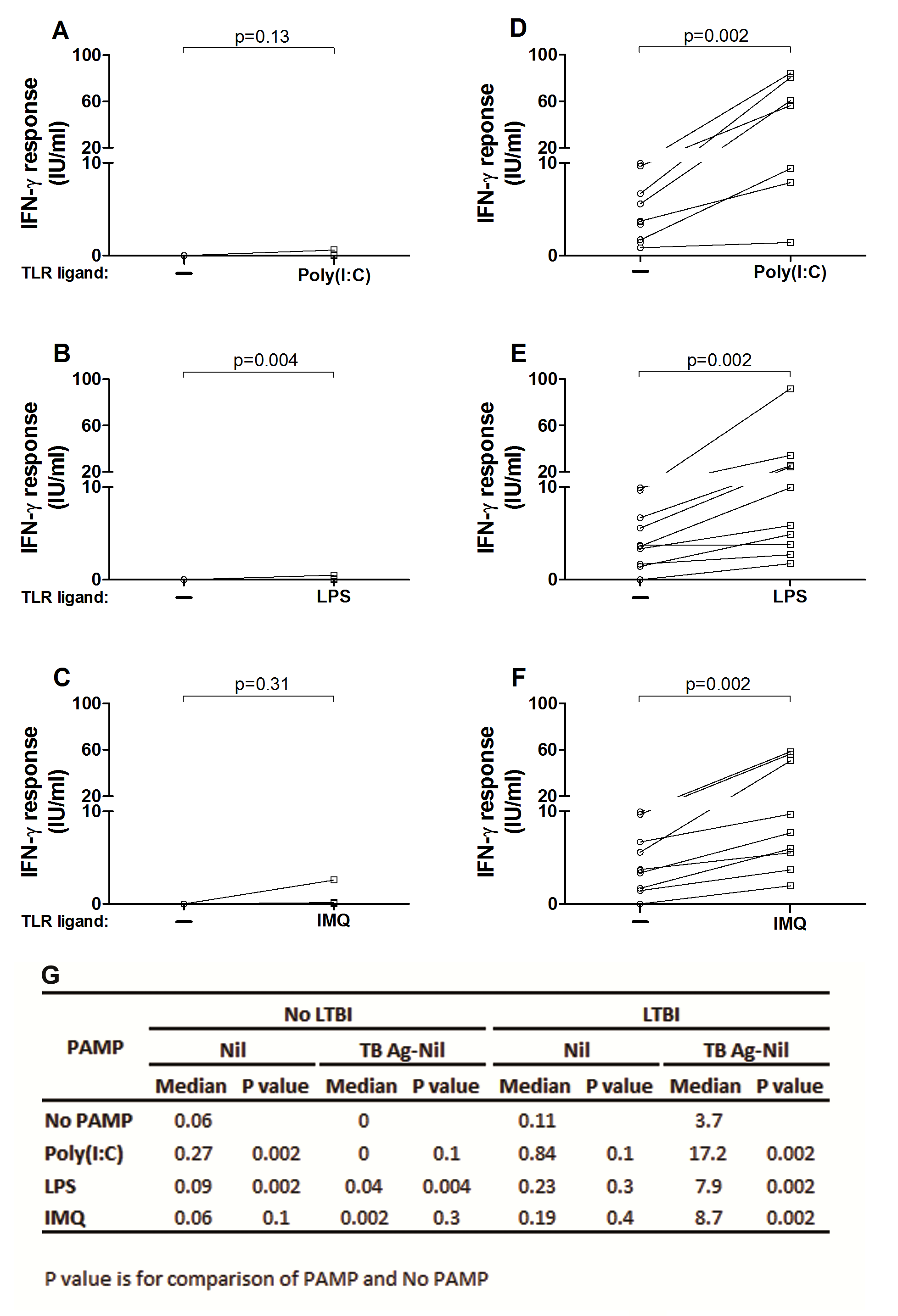

Supplement: Figure S2 — Immunomodulation of Quantiferon assay enhances the IFN-γ response in subjects with LTBI. IFN-γ response (TB Ag minus Nil) for ten uninfected controls (Panel A–C) and ten individuals with LTBI (Panel D–F) tested with the QFT-GIT assay in the absence or presence of poly(I:C) 40 µg/ml (Panel A,D), LPS 250 pg/ml (Panel B,E), and imiquimod (IMQ) 2 µg/ml (Panel C,F). Panel G shows comparison of modulated and unmodulated QFT-GIT results from infected and uninfected subjects. The Wilcoxon signed-rank test of medians, was used to compare paired modulated and unmodulated results from infected and uninfected subjects. (TIF) [file pone.0048027.s002.tif]

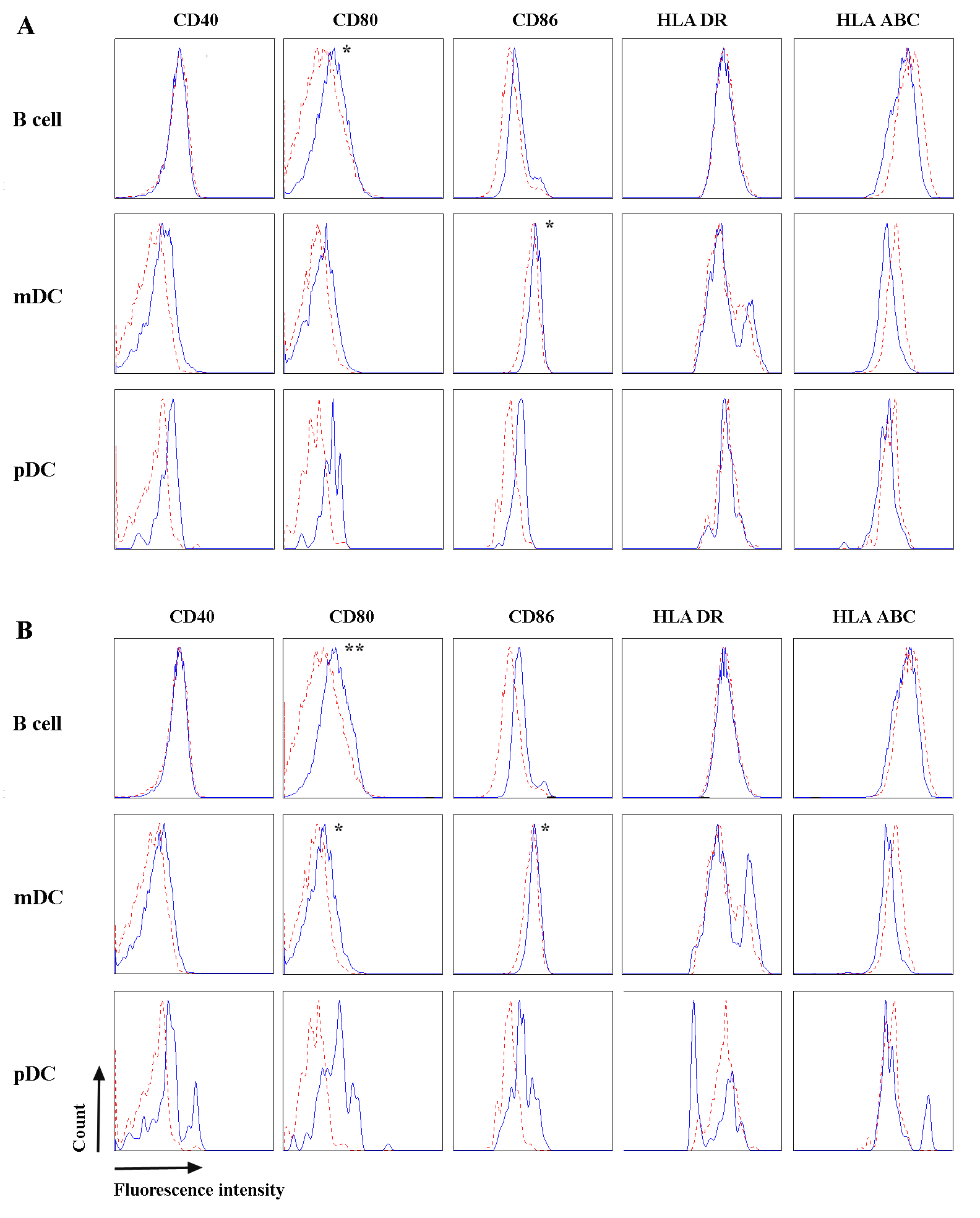

Supplement: Figure S3 — Flow cytometry analysis of surface expression of MHC and costimulatory molecules on B cells and dendritic cells stimulated with poly(I:C) and LPS. Whole blood was incubated in the QFT-GIT Nil tube in the absence (dashed red line) or presence (solid blue line) of poly(I:C) 40 µg/ml (Panel A) and LPS 250 pg/ml (Panel B) for 3 h. mDC, myeloid dendritic cell; pDC, plasmacytoid dendritic cell. The Wilcoxon signed-rank test was used to compare responses with and without PRR ligands. The asterisks indicate significant difference. *, P≤0.05, **, P≤0.005, *** P≤0.0005. (TIF) [file pone.0048027.s003.tif]
